# Association of Risk Factors With Patient-Reported Voice and Speech Symptoms Among Long-term Survivors of Oropharyngeal Cancer

**DOI:** 10.1001/jamaoto.2021.0698

**Published:** 2021-05-06

**Authors:** Puja Aggarwal, Katherine A. Hutcheson, Adam S. Garden, Frank E. Mott, Ryan P. Goepfert, Amber Duvall, Clifton D. Fuller, Stephen Y. Lai, G. Brandon Gunn, Erich M. Sturgis, Ehab Y. Hanna, Sanjay Shete

**Affiliations:** 1 Department of Epidemiology, The University of Texas MD Anderson Cancer Center, Houston; 2 Department of Head and Neck Surgery, The University of Texas MD Anderson Cancer Center, Houston; 3 Department of Radiation Oncology, The University of Texas MD Anderson Cancer Center, Houston; 4 Department of Thoracic Head and Neck Medical Oncology, The University of Texas MD Anderson Cancer Center, Houston; 5 Department of Otolaryngology–Head and Neck Surgery, Baylor College of Medicine, Houston, Texas; 6 Department of Biostatistics, The University of Texas MD Anderson Cancer Center, Houston; 7 Division of Cancer Prevention and Population Sciences, The University of Texas MD Anderson Cancer Center, Houston

## Abstract

**Importance:**

Voice and speech production are critical physiological functions that affect quality of life and may deteriorate substantially after oropharyngeal cancer (OPC) treatment. There is limited knowledge about risk factors associated with voice and speech outcomes among survivors of OPC.

**Objective:**

To identify the risk factors of voice and speech symptoms among long-term survivors of OPC.

**Design, Setting, and Participants:**

This retrospective cohort study with cross-sectional survivorship survey administration includes cancer-free survivors of OPC who were treated curatively between January 2000 and December 2013 at MD Anderson Cancer Center (Houston, Texas) who participated in a survey from September 2015 to July 2016. Of 906 survivors of OPC with a median survival duration at time of survey of 6 years (range, 1-16 years), patient-rated voice and speech outcomes for 881 were available and analyzed. The data were analyzed from June 30, 2020, to February 28, 2021.

**Main Outcomes and Measures:**

The primary outcome variable was patient-reported voice and speech scores that were measured using the MD Anderson Symptom Inventory–Head and Neck Cancer Module. Voice and speech scores of 0 to 4 were categorized as none to mild symptoms, and scores of 5 to 10 were categorized as moderate to severe symptoms. Risk factors for moderate to severe voice and speech symptoms were identified by multivariable logistic regression.

**Results:**

Among 881 survivors of OPC (median [range] age, 56 [32-84] years; 140 women [15.5%]; 837 White [92.4%], 17 Black [1.9%], and 35 Hispanic individuals [3.8%]), 113 (12.8%) reported moderate to severe voice and speech scores. Increasing survival time (odds ratio [OR], 1.17; 95% CI, 1.06-1.30) and increasing total radiation dose (OR, 1.16; 95% CI, 1.00-1.34), Black race (OR, 3.90; 95% CI, 1.02-14.89), Hispanic ethnicity (OR, 3.74; 95% CI, 1.50-9.35), current cigarette smoking at the time of survey (OR, 3.98; 95% CI, 1.56-10.18), treatment with induction and concurrent chemotherapy (OR, 1.94; 95% CI, 1.06-3.57), and late (OR, 7.11; 95% CI, 3.08-16.41) and baseline lower cranial neuropathy (OR, 8.70; 95% CI, 3.01-25.13) were risk factors associated with moderate to severe voice and speech symptoms. Intensity-modulated radiotherapy split-field regimen (OR, 0.31; 95% CI, 0.12-0.80; *P* = .01) was associated with lower likelihood of moderate to severe voice and speech symptoms.

**Conclusions and Relevance:**

This large OPC survivorship cohort study identified many treatment-related factors, including increasing total radiotherapy dose, multimodality induction and concurrent chemotherapy regimens, and continued smoking, as well as clinical and demographic factors, as risk factors that were associated with moderate to severe voice and speech symptoms. The key findings in this study were the protective associations of split-field radiation and that longer-term survivors, and those who continued to smoke, had worse voice and speech symptoms. These findings may inform research and effective targeted clinical voice and speech preservation interventions and smoking cessation interventions to maximize voice and speech function and address quality of life among patients with OPC.

## Key Points

**Question:**

What factors are associated with moderate to severe voice and speech symptoms among long-term survivors of oropharyngeal cancer (OPC)?

**Findings:**

In this retrospective cohort study with cross-sectional survivorship survey administration, of 881 survivors of OPC who were included in analysis, 113 (12.8%) reported moderate to severe voice and speech symptoms. Increasing survival time and total radiation dose, Black race, Hispanic ethnicity, current cigarette smoking at the time of the survey, multimodality treatment with induction and concurrent chemotherapy, and late and baseline lower cranial neuropathy were identified as risk factors for moderate to severe voice and speech symptoms, and an intensity-modulated split-field radiotherapy regimen was associated with better voice and speech symptoms.

**Meaning:**

These findings may have clinical implications for OPC treatment and survivorship, and the preservation of function and quality of life should be considered without compromising oncological outcomes.

## Teaser

This cross-sectional study examines risk factors of voice and speech symptoms among survivors of long-term oropharyngeal cancer.

## Introduction

Voice and speech production are complex physiological functions that are critical for verbal communication and social interaction and an inherent part of a person’s individuality and psychological well-being.^1^ It is important to distinguish that the voice is the production of sound by the larynx and speech is the articulation of that sound, primarily by tongue movement within the oral cavity and the lips. Patients with oropharyngeal cancer (OPC) typically receive radiotherapy (RT) and/or surgery, both of which affect the oral cavity, pharynx, and larynx, and can result in alterations of voice or speech production.^1^ Lingual injury from surgery or radiation can alter articulation and speech. Salivary alterations that are common after RT can also contribute to this dysfunction. Irradiation fields often include the larynx; therefore, RT-associated voice problems may occur because of altered vocal fold vibration, insufficient glottic closure, laryngeal mucosal dryness, and anatomical vocal tract changes, including edema, muscular atrophy, fibrosis, hyperemia, and erythema.^2^ Furthermore, oropharynx cancers or their treatment can affect speech via the hypoglossal nerve and the voice by primary tumor extension or delayed vagal nerve palsy after radiation.

According to a systematic review, patients with head and neck cancer (HNC) can experience some voice and speech dysfunction before any cancer treatment.^3^ Subsequent deterioration in these functions is expected as acute toxic effects of treatment immediately after treatment, with a predictable recovery up to 1 year after treatment completion.^3^ Approximately half of patients with OPC and HNC experience speech dysfunction posttreatment.^2,4^ In a study of long-term survivors of advanced HNC, 64% of patients treated with chemoradiotherapy (CRT) had abnormal speech scores, and a moderate to strong association was seen between automatic voice and speech assessments and more subjective perceptual outcome scores.^2^ A separate study identified voice and speech problems as among the top 5 symptoms reported by long-term survivors of OPC.^5^ Such impairment, including difficulty in speech articulation, suboptimal voice quality or resonance, and/or diminished intelligibility speech, may significantly affect daily function, social communications, and psychological well-being and lead to diminished quality of life (QoL).

A systematic review of patients with oral cancer and OPC who were treated from January 2000 to December 2008 demonstrated that age, subsite, primary tumor size, tumor node metastases stage, and comorbidities may affect speech posttreatment.^1^ Patients with advanced stage cancers, which tend to be treated more aggressively with multimodality treatment, may have worse speech symptoms than patients with early-stage tumors.^1^ Further, older patients with reduced physiologic reserve or comorbidities may take longer to recover or may not recover to the same extent as younger patients from the effects of cancer treatment on voice and speech function. Additionally, patients who require surgical resection of tumors in the oropharynx, especially as a salvage procedure after radiation, are likely to report worse voice and speech outcomes.^1^ Lastly, multimodality HNC treatment^1^ and late effects, such as lower cranial neuropathy (LCNP), can contribute to worse communication impairment.^5,6^

To our knowledge, few studies have identified predictors of voice and speech symptoms among patients with OPC. Most of these studies have focused on laryngeal cancer, in which the primary tumor itself may result in such impairments.^7,8^ Therefore, the aim of this study was to investigate clinical and demographic risk factors for patient-reported voice and speech impairment among long-term survivors of OPC. We hypothesized that tumor subsite, T-stage, multimodality treatment, RT dose, and LCNP would be associated with voice and speech symptom severity scores. The results from this study could inform the development of targeted voice and speech-preserving interventions in patients, as well as future research to optimize communication function among survivors of OPC.

## Methods

### Study Population

A survivorship survey, which was approved by the institutional review board, was collected from survivors of OPC who were treated at the University of Texas MD Anderson Cancer Center during January 1, 2000, to December 31, 2013. The survey was administered from September 9, 2015, to July 7, 2016, to 1740 survivors of OPC. Of these, 972 responded, providing a 56% response rate. All eligible adult (age at diagnosis ≥18 years) participants completed curative OPC treatment at least 1 year before survey collection and provided written informed consent to participate in the study. As our cohort eligibility required a minimum of 1-year disease-free survival time, these patients were referred to as *long-term survivors*.^5,6^

Patients who had died and those with diagnoses of locoregional recurrence, regional recurrence, distant metastasis, or secondary primary cancers of the head and neck, central nervous system, or thorax were excluded. Patients who declined participation and those who did not speak English also were excluded. Of the 972 survey respondents, 65 were excluded from the analyses because of having second primary malignant tumors, locoregional recurrence, or distant metastasis. One patient was excluded for not filling out any questions on the MD Anderson Symptom Inventory Head and Neck Cancer Module (MDASI-HN).

### Survey Instrument

The MDASI-HN is a validated multiple-symptom patient-reported outcome assessment questionnaire.^9,10,11,12^ This instrument includes 28 items, including the core component, which measures 13 symptoms that are common across all cancers; the head-neck–specific component, which measures 9 head and neck–specific localized symptoms; and the interference component, which includes 6 items that measure how symptoms affect daily function and activities.^9,10,11,12^ These items are rated on a scale of 0 (not present) to 10 (“as bad as you can imagine”).^9,10,11,12^ The head and neck component of the MDASI-HN asks questions regarding voice/speech difficulties, dry mouth, difficulty chewing/swallowing, choking/coughing, mucus in the mouth and throat, taste problems, skin pain/burning/rash, problems with teeth or gums, and constipation.^12^

### Primary Outcome

Cancer treatment–related patient-reported voice and speech symptoms representing global communication symptom as measured by the voice and speech question on the MDASI-HN was the primary outcome variable for this study. The format for MDASI-HN questions is as follows: “How severe are your symptoms? People with cancer frequently have symptoms that are caused by their disease or their treatment. We ask you to rate how severe the symptoms have been in the last 24 hours.” As part of the head-neck–specific component, patients were asked to rate their “difficulty with voice/speech at its worst.”^9^ Using previously published thresholds, voice and speech symptom scores were dichotomized based on response, with scores from 0 to 4 categorized as none to mild and scores from 5 to 10 categorized as moderate to severe.^10^

### Clinical and Sociodemographic Data Collection

Given the previous lack of knowledge of risk factors associated with voice and speech in OPC, we wanted to identify which of the clinicodemographic factors were associated with voice and speech. Therefore, our study included the following independent variables: clinical factors, treatment-related factors, and conditions such as late and baseline LCNP. We also included demographic factors, such as race/ethnicity, to account for and better understand their association with voice and speech symptoms. The electronic medical records of the study participants were reviewed for these data, as listed in Table 1. Staging was completed using the American Joint Committee on Cancer (AJCC), seventh edition. Survival time was estimated as the difference between age at the time of the survey and age at OPC diagnosis and represented the number of years a patient survived after OPC diagnosis. Cigarette smoking status was determined as follows: participants who had not smoked 100 cigarettes in their lifetime were classified as never smokers, those who had quit more than 6 months before diagnosis were considered former smokers at the time of diagnosis,^13,14^ and current smokers at the time of diagnosis were further categorized into those who quit subsequently and those who continued to smoke at time of the survey. Treatment modality comprised single modality treatment, which included patients who received only RT or only surgery, and multimodality treatment, which included patients who received combination treatment with CRT and those who received surgery followed by adjuvant RT or CRT. Chemotherapy, any induction chemotherapy, any concurrent chemotherapy, and combination treatment with induction and concurrent chemotherapy were assessed as yes/no variables. As in our previous publication, late LCNP was defined as cranial neuropathy of glossopharyngeal nerve (CN IX), vagus nerve (CN X), and hypoglossal nerve (CN XII), with onset 3 months or more post–cancer therapy and baseline LCNP, including LCNP onset before cancer therapy.^5^

**Table 1.** Characteristics of 906 Patients With OPC and Distribution by Clinical and Sociodemographic Variables and Voice and Speech Symptom Categories

Variable	No. (%)			
	All patients with OPC	Voice and speech		
		Information missing	Symptom	
			None to mild	Moderate to severe
No.	906	25	768	113
Age at diagnosis, median (range), y	56 (32-84)	NA	56 (32-84)	55 (42-82)
Survival time, median (range), y^a^	6 (1-16)	NA	6 (1-16)	9 (2-16)
Radiation dose, median (range), Gy^a^	70 (40-72.6)	NA	66 (40-72)	70 (60-72.6)
Sex				
Women	140 (15.5)	5	117 (86.7)	18 (13.3)
Men	766 (84.6)	10	651 (87.3)	95 (12.7)
Education				
≥High school	650 (71.7)	17	556 (87.8)	77 (12.2)
<High school	171 (18.9)	5	143 (86.1)	23 (13.9)
Missing	85 (9.4)	3	69 (84.2)	13 (15.9)
Race/ethnicity^b^				
White	837 (92.4)	22	719 (88.2)	96 (11.8)
Black	17 (1.9)	1	11 (68.8)	5 (31.2)
Hispanic	35 (3.8)	2	24 (72.7)	9 (27.3)
Other	8 (0.9)	0	6 (75.0)	2 (25.0)
Missing	9 (1.0)	0	8 (88.9)	1 (11.1)
Primary site				
Tonsil	418 (46.2)	9	363 (88.8)	46 (11.2)
Base of tongue + GPS	456 (50.3)	16	377 (85.7)	63 (14.3)
Other	32 (3.5)	0	28 (87.5)	4 (12.5)
T category^b^				
Treatment, T1, T2	684 (75.5)	19	597 (89.8)	68 (10.2)
T 3, T4	222 (24.5)	6	171 (79.2)	45 (20.8)
N category^b^				
N0, N1, N2a, N2b	727 (80.2)	16	637 (89.6)	74 (10.4)
N2c, N3	179 (19.8)	9	131 (77.1)	39 (22.9)
HPV status^b^				
Negative	58 (6.4)	1	50 (87.7)	7 (12.3)
Positive	440 (48.6)	12	389 (90.9)	39 (9.1)
Unknown	408 (45.0)	12	329 (83.1)	67 (16.9)
Cigarette smoking^b^				
Never	420 (46.3)	13	361 (88.7)	46 (11.3)
Former smokers at time of diagnosis	343 (37.9)	7	296 (88.1)	40 (11.9)
Quit smoking subsequent to diagnosis	95 (10.5)	3	75 (81.5)	17 (18.5)
Current smoker at time of survey	36 (4.0)	2	24 (70.6)	10 (29.4)
Do not know	12 (1.3)	0	12 (100.0)	0
Solid food pretreatment^c^				
Yes	894 (98.7)	25	758 (87.2)	111 (12.8)
No	12 (1.3)	0	10 (83.3)	2 (16.7)
Treatment group^b^				
Single modality	280 (30.9)	8	249 (91.5)	23 (8.5)
Multimodality	626 (69.1)	17	519 (85.2)	90 (14.8)
Treatment modalities				
RT alone	272 (30.0)	8	241 (91.3)	23 (8.7)
Surgery alone	8 (0.9)	0	8 (100.0)	0
RT plus systemic	610 (67.3)	16	506 (85.2)	88 (14.8)
Surgery plus adjuvant	16 (1.8)	1	13 (86.7)	2 (13.3)
Chemotherapy^b^				
No	286 (31.6)	9	254 (91.7)	23 (8.3)
Yes	620 (68.4)	16	514 (85.1)	90 (14.9)
Surgery				
No	881 (97.2)	24	746 (87.1)	111 (12.9)
Yes, robotic	18 (2.0)	0	17 (94.4)	1 (5.6)
Yes, open	7 (0.8)	1	5 (83.3)	1 (16.7)
Neck dissection				
No	679 (74.9)	21	572 (86.9)	86 (13.1)
Yes	227 (25.1)	4	196 (87.9)	27 (12.8)
RT				
No	8 (0.9)	0	8 (100.0)	0
Yes	898 (99.1)	25	760 (87.1)	113 (12.9)
RT schedule^d^^,^^b^				
Standard fractionation	798 (88.1)	21	691 (88.9)	86 (11.1)
Accelerated	100 (11.0)	4	69 (71.9)	27 (28.1)
Missing/no RT	8 (0.9)	0	8 (100.0)	0
RT type^e^^,^^b^				
3D-CRT	51 (5.6)	3	27 (56.3)	21 (43.7)
IMRT-SF	677 (74.7)	14	587 (88.5)	76 (11.5)
IMRT-WF +VMAT	48 (5.3)	1	38(80.9)	9 (19.1)
Proton	22 (2.4)	1	18 (85.7)	3 (14.3)
IMRT ipsilateral	100 (11.0)	6	90 (95.7)	4 (4.3)
Missing/no	8 (0.9)	0	8 (100.0)	0
Combination induction and concurrent chemotherapy^b^				
No	739 (81.6)	18	641 (88.9)	80 (11.1)
Yes	167 (18.4)	7	127 (79.4)	33 (20.6)
Induction chemotherapy only				
No	610 (67.3)	16	526 (88.6)	68 (11.4)
Yes	296 (32.7)	9	242 (84.3)	45 (15.7)
Concurrent chemotherapy only^b^				
No	419 (46.3)	12	370 (90.9)	37 (9.1)
Yes	487 (53.7)	13	398 (84.0)	76 (16.0)
Late LCNP^b^				
No	869 (95.9)	24	751 (88.9)	94 (11.1)
Yes	37 (4.1)	1	17 (47.2)	19 (52.8)
Baseline LCNP^b^				
No	887 (97.9)	25	759 (88.0)	103 (12.0)
Yes	19 (2.1)	0	9 (47.4)	10 (52.6)

Abbreviations: 3D-CRT, 3-dimensional conformal radiotherapy; GPS, glossopharyngeal sulcus; HPV, human papillomavirus; IMRT-SF, intensity-modulated radiotherapy split-field technique; IMRT-WF, intensity-modulated radiotherapy whole-field technique; LCNP, late lower cranial neuropathy; OPC, oropharyngeal cancer; RT, radiotherapy; VMAT, volumetric-modulated arc therapy.

^a^ *P* < .05 for Kruskal-Wallis test.

^b^ *P* < .05 for Fisher exact test.

^c^ Solid food diet pretreatment was controlled for as a surrogate for pretreatment oral dysfunction/symptoms.

^d^ Radiotherapy fractionation schedule included standard fractionation (70.0 Gy given in 33-35 fractions), accelerated fractionation (72.0 Gy given in 40 fractions or concomitant boost or Danish Head and Neck Cancer Group RT regimens), and no RT.

^e^ Radiotherapy types included 3D-CRT, bilateral IMRT-SF, IMRT-WF, VMAT, proton therapy, and ipsilateral IMRT regimens.

### Statistical Analysis

Descriptive statistics were used to summarize the study population. The Kruskal-Wallis test and Fisher exact test were used to test for differences by voice and speech symptom subgroups for continuous and categorical variables, respectively. Univariate analysis estimated crude unadjusted associations, and multivariable logistic regression analysis was used to investigate associations between clinical and sociodemographic variables and moderate to severe voice and speech symptom scores. Age at diagnosis, subsite, T category, smoking status, and treatment modality were defined as a priori key clinical variables. Unadjusted univariate and adjusted multivariable odds ratios (ORs) and their 95% CIs were computed. Multicollinearity was assessed with the variance inflation factor being greater than 10. A 2-sided *P* ≤ .05 was considered statistically significant. Analysis was performed using Stata, version 14.0 (StataCorp).

## Results

### Sample Characteristics

A total of 906 survivors of OPC were included in this study. Of the 881 survivors of OPC who responded to the voice and speech symptom question with a median duration of time from diagnosis to survey of 6.0 years (range, 1-16 years), 113 (12.8%) reported moderate to severe voice and speech symptom scores and 288 (32.7%) reported mild voice and speech symptom scores. Table 1 summarizes the sample characteristics and all clinical and sociodemographic variables that were stratified by voice and speech symptom classifications. Median survival (median, 9 years [range, 2-16 years] vs median, 6 years [range, 1-16 years]) and median RT dose (median, 70.0 Gy [range, 60-72.6 Gy] vs median, 66.3 Gy [range, 40.0-72.0 Gy]) were significantly higher among survivors with moderate to severe voice and speech symptoms compared with those who reported none to mild voice and speech symptoms. Among those who self-identified as Black and Hispanic, 5 of 16 (31.2%) and 9 of 33 (27.3%), respectively, reported moderate to severe voice and speech symptoms. Thirty-three of 160 patients (20.6%) who received induction and concurrent chemotherapy regimens reported moderate to severe voice and speech symptoms. Further, among survivors with late LCNP and baseline LCNP, 19 of 37 (52.8%) and 10 of 19 (52.6%), respectively, reported moderate to severe voice and speech symptoms. Single-item voice and speech scores were positively correlated with single-item swallowing (Spearman ρ = 0.534), single-item choking/coughing (Spearman ρ = 0.498), and single-item dry mouth (Spearman ρ = 0.291) scores on the MDASI-HN. The frequency distribution of voice and speech symptom scores is reported in the eTable in the Supplement.

Table 2 summarizes the univariate and multivariable analyses of risk factors for moderate to severe voice and speech symptom scores. On multivariable logistic regression analysis, increasing survival time (OR, 1.17; 95% CI, 1.06-1.30), increasing total RT dose (OR, 1.16; 95% CI, 1.00-1.34), Black race (OR, 3.90; 95% CI, 1.02-14.89), Hispanic ethnicity (OR, 3.74; 95% CI, 1.50-9.35), current cigarette smoking at the time of the survey (OR, 3.98; 95% CI, 1.56-10.18), combined treatment with induction and concurrent chemotherapy (OR, 1.94; 95% CI, 1.06-3.57), late LCNP (OR, 7.11; 95% CI, 3.08-16.41), and baseline LCNP (OR, 8.70; 95% CI, 3.01-25.13) were identified as risk factors that were associated with increased odds of developing moderate to severe voice and speech symptoms after adjustment, whereas RT type, including intensity-modulated RT–split-field (IMRT-SF) regimen (OR, 0.31; 95% CI, 0.12-0.80) was associated with lower likelihood of moderate to severe voice and speech symptoms.

**Table 2.** Multivariable Logistic Regression Analysis Assessing the Association Between Clinical and Sociodemographic Variables and Patient-Reported Moderate to Severe Voice and Speech Symptoms

Variables	OR (95% CI)	
	Univariate	Multivariable
Age at diagnosis, y	1.00 (0.98-1.03)	1.03 (1.00-1.06)
Survival time, y^a^^,^^b^	1.12 (1.07-1.18)	1.17 (1.06-1.30)
Radiation dose, Gy^a^^,^^b^	1.29 (1.17-1.41)	1.16 (1.00-1.34)
Sex		
Men	1 [Reference]	1 [Reference]
Women	1.05 (0.61-1.81)	1.15 (0.62-2.16)
Education new		
≥High school	1 [Reference]	1 [Reference]
<High school	1.16 (0.70-1.92)	1.02 (0.56-1.84)
Race/ethnicity		
Non-Hispanic^a^^,^^b^		
White	1 [Reference]	1 [Reference]
Black	3.40 (1.16-10.01)	3.90 (1.02-14.89)
Hispanic individuals	2.81 (1.27-6.22)	3.74 (1.50-9.35)
Other	2.50 (0.50-12.54)	3.01 (0.46-19.82)
Subsite		
Tonsil	1 [Reference]	1 [Reference]
Base of tongue + GPS	1.32 (0.88-1.98)	1.18 (0.71-1.98)
Others	1.13 (0.38-3.36)	1.00 (0.28-3.59)
T category		
1 + 2	1 [Reference]	1 [Reference]
3 + 4^a^	2.31 (1.53-3.49)	0.71 (0.39-1.28)
N category		
N0	1 [Reference]	1 [Reference]
N2c+N3^a^	2.56 (1.67-3.94)	1.30 (0.76-2.25)
HPV status		
Negative	1 [Reference]	1 [Reference]
Positive	0.72 (0.30-1.69)	1.15 (0.42-3.15)
Unknown	1.45 (0.63-3.35)	1.17 (0.43-3.18)
Cigarette smoking		
Never	NA	1 [Reference]
Former smokers at time of diagnosis	1.06 (0.68-1.66)	1.02 (0.60-1.72)
Quit smoking subsequent to diagnosis	1.78 (0.97-3.27)	1.69 (0.82-3.48)
Current smoker at time of survey^a^^,^^b^	3.27 (1.47-7.27)	3.98 (1.56-10.18)
Solid food pretreatment		
No	1 [Reference]	1 [Reference]
Yes	0.73 (0.16-3.39)	2.43 (0.28-20.78)
Treatment group		
Single modality	1 [Reference]	1 [Reference]
Multimodality^a^	1.88 (1.16-3.04)	1.85 (0.92-3.71)
Surgery		
No	1 [Reference]	1 [Reference]
Yes, robotic	0.40 (0.05-3.00)	0.99 (0.08-12.20)
Yes, open	1.34 (0.16-11.61)	1.16 (0.08-18.03)
Neck dissection		
No	1 [Reference]	1 [Reference]
Yes	0.92 (0.58-1.45)	0.87 (0.50-1.51)
**RT**		
Schedule		
Standard fractionation	1 [Reference]	1 [Reference]
Accelerated^a^	3.14 (1.91-5.17)	0.71 (0.29-1.76)
Type		
3D conformal	1 [Reference]	1 [Reference]
IMRT-SF^a^^,^^b^	0.17 (0.09-0.31)	0.31 (0.12-0.80)
IMRT-WF + VMAT^a^	0.30 (0.12-0.77)	0.61 (0.17-2.17)
Proton^a^	0.21 (0.06-0.83)	0.60 (0.11-3.26)
IMRT ipsilateral^a^	0.06 (0.02-0.18)	0.25 (0.06-1.08)
Induction and concurrent chemotherapy		
No	1 [Reference]	1 [Reference]
Yes^a^^,^^b^	2.08 (1.33-3.26)	1.94 (1.06-3.57)
**LCNP**		
Late		
No	1 [Reference]	1 [Reference]
Yes^a^^,^^b^	8.93 (4.49-17.78)	7.11 (3.08-16.41)
Baseline		
No	1 [Reference]	1 [Reference]
Yes^a^^,^^b^	8.19 (3.25-20.62)	8.70 (3.01-25.13)

Abbreviations: 3D-CRT, 3-dimensional conformal radiotherapy; GPS, glossopharyngeal sulcus; HPV, human papillomavirus; IMRT-SF, intensity-modulated radiotherapy split-field technique; IMRT-WF, intensity-modulated radiotherapy whole-field technique; LCNP, late lower cranial neuropathy; OR, odds ratio; RT, radiotherapy; VMAT, volumetric-modulated arc therapy.

^a^ *P* < .05 after univariate analysis.

^b^ *P* < .05 after multivariable analysis.

## Discussion

This large OPC survivorship study provides a comprehensive assessment of risk factors that are associated with moderate to severe patient-reported voice and speech symptoms in long-term follow-up. To our knowledge, this study is the first to identify demographic and treatment-related risk factors that are associated with moderate to severe voice and speech symptoms. These factors included increasing the total RT dose, combined induction and concurrent chemotherapy regimens, increasing survival time, current cigarette smoking status at the time of the survey, Black race, and Hispanic ethnicity. Neither salvage/post-RT nor up-front neck dissection was associated with severity of voice and speech symptoms. The association between late LCNP and moderate to severe voice and speech symptoms was expected, and the correlation of late LCNP and worse voice and speech symptom scores has been previously reported by our group.^5^ Similarly, the association between baseline LCNP and voice and speech symptoms was expected, as pretreatment damage to lower cranial nerves is associated with baseline voice and speech disturbance, which is likely to persist and often progress through treatment.

Modern IMRT regimens, specifically IMRT-SF, were associated with less severe voice and speech symptoms. A key finding was the identification of increasing total RT dose to the primary OPC tumor site as a significant risk factor that was associated with moderate-to-severe voice and speech problems. On average, an increase in total RT dose by 1 Gy was associated with a 17% increase in the odds of moderate to severe voice and speech scores. This finding is plausible, given that higher total RT dose given to the primary tumor is likely to contribute to a higher radiation dose to the nearby larynx and subsequently cause greater laryngeal, voice, and speech dysfunction. Dornfeld et al^6^ demonstrated that irradiation of false vocal cords and pharyngeal wall to a dose greater than 66 Gy was associated with worse speech-related QoL scores, while Sanguineti et al^15^ reported a mean laryngeal dose of 50 Gy as a potential threshold to reduce grade 0 to 1 voice alterations among patients with OPC. Treatment-related soft tissue damage to the pharynx and larynx from radiation, as well as surgery, leads to fibrosis and atrophy along with disruptions in lymphatic drainage and salivary function. These effects are likely explanations for the voice and speech impairments that patients report. Furthermore, LCNPs, regardless of their etiology, are likely to significantly negatively affect patient-reported voice and speech function. Resonance disturbance as a consequence of soft palate injury from radiation fibrosis or surgery can cause velopharyngeal insufficiency and may also contribute to voice and speech impairment.

Consistent with other studies, we found that survivors of OPC who received more modern conformal RT regimens, such as IMRT-SF, were less likely than survivors who received older 3-dimensional conformal RT regimens to report moderate to severe voice and speech scores.^4,16,17^ Intensity-modulated RT regimens deliver a highly precise, conformal high RT dose to heterogeneously shaped tumor targets.^16^ Therefore, IMRT can prioritize or maximize sparing of anatomical structures critical for speech and voice, including the larynx, and sparing these structures may contribute to less vocal dysfunction and speech impairment.^4,16,17^

Treatment with induction chemotherapy followed by concurrent chemotherapy was associated with an increased risk of moderate to severe voice and speech impairment. As chemotherapy sensitizes tumor and adjacent tissues to the effects of radiation, it also exacerbates RT-associated adverse effects.^4^ Furthermore, CRT may negatively affect normal tissue mobility and subsequent voice and speech dysfunction.^6^ Morton et al^18^ reported significantly worse speech impairment among patients with HNC who were receiving multimodality treatment. Chemotherapy-associated cytotoxicity can also contribute to xerostomia and oral mucositis, which may lead to further decline in functional and speech outcomes.^4^ Further, the severity of short-term toxic effects has been associated with long-term outcomes; therefore, it is not surprising that more intense CRT may contribute to more severe voice and speech dysfunction over time.^1^

Continued cigarette smoking was also associated with increased risk of moderate to severe voice and speech symptoms and is consistent with a previous study.^19^ Heavy/extensive smoking may cause irritation and dryness of laryngeal mucosa, which leads to vocal cord inflammation and irritability, which in turn can contribute to cough, respiratory sputum, and voice alterations.^20^ Also, a metanalysis has shown that smoking negatively affects voice pitch, maximum phonation time, voice handicap index, and voice physical subfunctions/domains.^20^

Increased survival time was also associated with moderate to severe voice and speech scores in the current study. The literature suggests that voice and speech scores may improve on posttreatment follow-up; however, this was not the case in our study.^3^ Our results suggest that patients with longer survival may, as they get older, experience some accumulating late toxic effects of cancer treatment on voice and speech, including LCNP and age-related vocal changes, such as presbyphonia. Presbyphonia is associated with known anatomical changes to the larynx, including a reduction in elastic fibers and sarcopenia; these changes result in vocal fold atrophy, poor vocal projection/loudness, breathiness, and vocal roughness.^21,22^ Furthermore, longer survival may act as a proxy measure for older treatments that are known to have higher toxic effects.^1^ Lastly, our results are consistent with a previous study among patients with OPC that reported that follow-up time after cancer treatment was univariately associated with speech dysfunction.^2^

To our knowledge, our study is the first to identify that Black and Hispanic survivors OPC were more likely to report moderate to severe voice and speech symptoms, despite the small sample sizes of these subgroups. One might postulate differences in T category, RT dosage, RT type, education, or concurrent chemotherapy that may have contributed to increased dysphonia in Black and Hispanic survivors of OPC; however, this was not the case in our study. Our study results are plausible, given that previous literature demonstrates that Black individuals had worse voice outcomes vs White^23^ and Hispanic individuals, and Black individuals with voice dysfunction reported that they may delay medical care because of problems with access to health care.^24^ Further, the etiology of racial/ethnic differences in vocal dysfunction is multifactorial and needs to be elucidated in future studies. However, we postulate that the racial/ethnic differences observed in our study may be owing to low income, lack of health insurance, lack of awareness, differences in the perception of symptoms, behavioral/lifestyle choices, cultural and social differences, and possibly other environmental factors or other comorbidities.^23,24^

### Limitations

Among the limitations of our study is that a single question from the validated patient-reported MDASI-HN was used to assess severity of voice and speech impairment without objective, clinician-determined assessments of voice and speech production. Nevertheless, patient-reported functional outcomes provide more precise, patient-specific information, as physician-rated voice dysfunction may underestimate patient-rated voice problems.^25^ Data on pretreatment voice and speech scores, resonance disorders, and other possible associated comorbidities were not available. We also could not separate speech and voice for the outcomes in our study. Future prospective studies that investigate voice and speech function in patients with OPC would ideally include baseline measurements; objective assessments, such as videostroboscopy, articulation metrics, and voice acoustic analysis; and more granular measurements of patient-reported voice and speech impairment. Audioperceptual evaluation and physical examination with direct visualization of oral cavity, palate, and vocal fold functioning would also be necessary to help identify the pathophysiology underlying self-reported symptoms. Another possible limitation of our study is survival bias, which we tried to address by controlling for survival time in our multivariable model. Also, disease in our study was staged using the AJCC seventh edition; therefore, our study findings may not be generalizable to the current AJCC eighth edition staging criteria. In our data, the number of patients who had surgery was low; therefore, our results may not be generalizable to surgically treated patients. Lastly, specific treatment data, including radiation field dosimetric data and respective dose constraints, were not available for our entire cohort and should be investigated in future longitudinal studies.

## Conclusions

Our study identified several clinical factors that are associated with patient-reported voice and speech impairment among a large cohort of survivors of OPC. The key finding in this study was the protective association of SF radiation technique, which was a laryngeal dose–sparing RT planning technique that was popular at our institution during the study period. Furthermore, longer-term survivors and those who continued to smoke had worse outcomes. Smoking cessation efforts continue to be needed in the realm of OPC survivorship. Given the ever-growing population of comparatively healthy patients with human papillomavirus–associated OPC who are likely to survive decades after treatment, our therapeutic efforts to preserve function and QoL are important but should not compromise oncological outcomes. In situations in which these goals are competing, they should be prioritized and consider individual patient preferences and priorities through a shared decision-making process with the patient and their families.

## Notes

Supplement. eTable. Frequency distribution of voice and speech symptom scores (N=906)
