# Supplementary material for: Association of Risk Factors With Patient-Reported Voice and Speech Symptoms Among Long-term Survivors of Oropharyngeal Cancer
Source: JAMA Otolaryngol Head Neck Surg. 2021 May 6;147(7):1–9. doi: 10.1001/jamaoto.2021.0698 (PMC8103354; doi:10.1001/jamaoto.2021.0698)
Supplement: Supplement. — eTable. Frequency distribution of voice and speech symptom scores (N=906) [file jamaotolaryngolheadnecksurg-e210698-s001.pdf]

Supplemental Online Content

Aggarwal P, Hutcheson KA, Garden AS, et al. Association of risk factors with patient-reported voice and speech symptoms among long-term survivors of oropharyngeal cancer. *JAMA Otolaryngol Head Neck Surg*. Published online May 6, 2021. doi:10.1001/jamaoto.2021.0698

eTable. Frequency distribution of voice and speech symptom scores (N=906)

This supplemental material has been provided by the authors to give readers additional information about their work.

© 2021 Aggarwal P et al. *JAMA Otolaryngology–Head & Neck Surgery.*

eTable. Frequency distribution of voice and speech symptom scores (N=906)

MDASI-HN Voice/Speech Score	Number of Patients	Proportion
**0**	480	0.54
**1**	132	0.15
**2**	88	0.10
**3**	45	0.05
**4**	23	0.03
**5**	36	0.04
**6**	13	0.01
**7**	22	0.02
**8**	21	0.02
**9**	5	0.01
**10**	16	0.02
**Missing**	25	0.03

Abbreviations: MDASI-HN; MD Anderson Symptom Inventory - Head and Neck Cancer Module

© 2021 Aggarwal P et al. *JAMA Otolaryngology–Head & Neck Surgery.*
